# Multi-State Recognition of Electro-Hydraulic Servo Fatigue Testers via Spatiotemporal Fusion and Bidirectional Cross-Attention

**DOI:** 10.3390/s25237229

**Published:** 2025-11-26

**Authors:** Guotai Huang, Shuang Bai, Xiuguang Yang, Xiyu Gao, Peng Liu

**Affiliations:** 1 School of Mechanical and Aerospace Engineering, Jilin University, Changchun 130022, China; 2 Sinotest Equipment Co., Ltd., Changchun 130012, China

**Keywords:** multi-task learning, electro-hydraulic servo fatigue testing machine, spatiotemporal feature fusion, bidirectional cross-attention, state recognition

## Abstract

Electro-hydraulic servo fatigue testing machines are susceptible to concurrent degradation and failure of multiple components during high-frequency, high-load, and long-duration cyclic operations, posing significant challenges for online health monitoring. To address this, this paper proposes a multi-state recognition method based on spatiotemporal feature fusion and bidirectional cross-attention. The method employs a Bidirectional Temporal Convolutional Network (BiTCN) to extract multi-scale local features, a Bidirectional Gated Recurrent Unit (BiGRU) to capture forward and backward temporal dependencies, and Bidirectional Cross-Attention (BiCrossAttention) to achieve fine-grained bidirectional interaction and fusion of spatial and temporal features. During training, GradNorm is introduced to dynamically balance task weights and mitigate gradient conflicts. Experimental validation was conducted using a real-world multi-sensor dataset collected from an SDZ0100 electro-hydraulic servo fatigue testing machine. The results show that on the validation set, the cooler and servo valve achieved both accuracy and F1-scores of 100%, the motor-pump unit achieved an accuracy of 98.32% and an F1-score of 97.72%, and the servo actuator achieved an accuracy of 96.39% and an F1-score of 95.83%. Compared to single-task models with the same backbone, multi-task learning improved performance by approximately 3% to 4% for the hydraulic pump and servo actuator tasks, while significantly reducing overall deployment resources. Compared to single-task baselines, multi-task learning improves performance by 3–4% while reducing deployment parameters by 75%. Ablation studies further confirmed the critical contributions of the bidirectional structure and individual components, as well as the effectiveness of GradNorm in multi-task learning for testing machines, achieving an average F1-score of 98.38%. The method also demonstrated strong robustness under varying learning rates and resampling conditions. Compared to various deep learning and fusion baseline methods, the proposed approach achieved optimal performance in most tasks. This study provides an effective technical solution for high-precision, lightweight, and robust online health monitoring of electro-hydraulic servo fatigue testing machines under complex operating conditions.

## 1. Introduction

A material fatigue testing machine is a significant device used to evaluate the fatigue performance of materials under long-term cyclic loading [1]. As fundamental equipment in manufacturing, it is widely employed in cutting-edge fields such as aerospace, defense systems, and material research. With the increasingly complex structures of modern high-end equipment, some engineering components now require fatigue life spans of 10^9^ to 10^12^ cycles, making fatigue testing demands more stringent. Electro-hydraulic servo material fatigue testing machines, capable of applying high loads and various stress types, are extensively used in dynamic high-cycle fatigue, low-cycle fatigue, program-controlled fatigue, and conventional mechanical property testing scenarios [2]. These machines operate under complex alternating loads for prolonged periods, making key components (e.g., actuators, servo valves, seals, sensors) prone to performance degradation and failures, leading to distorted test data, specimen loss, and even safety incidents. Concurrently, fatigue tests now involve longer loading cycles, higher frequencies, and rising costs, intensifying the need for efficient, stable, and reliable performance under high-cycle and heavy-load conditions [3]. Furthermore, according to industry research, over 60% of unplanned downtime is caused by the failure of multiple components working together. However, existing commercial systems still rely on manual inspections or isolated threshold alarms, lacking the ability to intelligently perceive the concurrent evolution of multiple states. Thus, health monitoring for electro-hydraulic servo fatigue testing machines is critically important. By collecting and analyzing operational parameters to assess health status, downtime losses can be reduced, equipment lifespan extended, and testing efficiency improved, ultimately lowering experimental costs. Consequently, the development of high-precision, real-time intelligent health monitoring approaches holds substantial theoretical and practical significance in industrial applications [4].

In recent years, with big data and artificial intelligence technologies increasingly applied in industry, material fatigue testing machines have evolved toward informatization and intelligence. Big data technologies overcome the limitations of traditional spot-check data, enabling effective collection and processing of operational data [5]. Advances in AI allow systems to use intelligent algorithms to extract fault patterns and operational rules from data [6]. These innovations provide robust technical support for health monitoring, propelling electro-hydraulic servo fatigue testing equipment into the era of data-driven condition monitoring, while also posing major challenges for real-time, accurate monitoring of key components [7].

Data-driven condition monitoring research has expanded across various electromechanical equipment domains [8]. Yan et al. reviewed the application and trends of Continuous Wavelet Transform (CWT), Discrete Wavelet Transform (DWT), and Wavelet Packet Transform (WPT) in rotating machinery fault diagnosis [9].

Recent years have seen rapid development in deep learning for condition perception in electromechanical–hydraulic systems [10]. 1D-CNNs automatically extract local temporal patterns from multi-source sequences like vibration, pressure, flow, and valve current [11]. RNN/LSTM/GRU models excel at capturing long-term dependencies, while TCNs use dilated convolutions to achieve large receptive fields with parallel processing and stable gradients [12,13]. Attention mechanisms and Transformer architectures further enhance selectivity for non-stationary, strongly coupled signals and global modeling capabilities [14,15,16].

In hydraulics and servo systems, studies have applied 1D-CNN/TCN or CNN-RNN hybrid networks for valve fault identification, leakage and seal degradation detection, and actuator friction/clearance anomaly diagnosis, incorporating multi-sensor early/mid/late feature fusion to improve robustness [17]. Tang et al. proposes an intelligent fault diagnosis method based on deep learning and Bayesian optimization for fault detection and analysis in hydraulic piston pumps [18]. Zhang et al. proposes a fault diagnosis method for electro-hydraulic servo systems based on a novel 1DCNN-LSTM architecture integrated with attention mechanisms and transfer learning, aiming to enhance the accuracy and efficiency of fault diagnosis [19]. However, simple concatenation or weighted fusion often fails to explicitly model the driving relationships and phase differences among pressure, displacement, current, and force signals. Single-task learning is also susceptible to sample imbalance and label noise, limiting generalization under variable operating conditions and loading frequencies.

Multitask learning (MTL) offers an effective approach for equipment health management. By sharing temporal representations and jointly performing subtasks like fault type identification, fault severity/degradation index regression, operating condition recognition, and even remaining useful life (RUL) estimation, MTL enables information complementarity and parameter regularization, mitigating small-sample and class-imbalance issues [20]. Meanwhile, cross-attention mechanisms for multi-source sequences can adaptively align temporal dependencies and phase characteristics across different channels while highlighting critical measurement points and frequency bands, enhancing the physical consistency of multimodal coupled representations [21]. To address limitations like traditional RNN gradient decay, pure CNN inadequacy in capturing long-range dependencies, causal TCN inability to utilize “future” context, and standard attention neglect of cross-channel interactions, recent trends favor bidirectional temporal-aware network structures and refined cross-modal attention to strengthen spatiotemporal coupling modeling capabilities [22].

Although numerous methods have achieved satisfactory application outcomes in their respective domains, several limitations remain:

(1) While data-driven approaches have made notable progress in the monitoring of complex electromechanical equipment, research on state recognition specifically for fatigue testing machines is still scarce. Moreover, most existing studies focus on single-task learning, whereas in industrial practice, enabling a single model to perform multi-task monitoring can substantially reduce deployment resources, labor costs, and time expenditure.

(2) The majority of existing studies employ unidirectional sequence models, which limits the exploitation of both past and future contextual information. Research on the integration of inter-sensor features and temporal characteristics remains insufficient, and most attention mechanisms are restricted to unidirectional or single-stream designs.

(3) In multitask learning, issues such as task imbalance and gradient conflicts are frequently overlooked. Although dynamic weighting strategies (e.g., GradNorm) have been proposed, their application in industrial testing equipment scenarios is still limited.

To address these challenges, this work proposes a multi-state monitoring approach based on spatiotemporal feature fusion and a bidirectional cross-attention mechanism. Specifically, a Bidirectional Temporal Convolution Network (BiTCN) is employed to capture multi-scale temporal information, which is further modeled using a Bidirectional Gated Recurrent Unit (BiGRU) to represent sequential dependencies in both forward and backward directions. During the feature fusion stage, a Bidirectional Cross-Attention (BiCrossAttention) mechanism is introduced to facilitate fine-grained information exchange and selection between different channels and tasks. In the multitask training process, the GradNorm strategy is adopted to dynamically adjust task loss weights, thereby mitigating gradient conflicts and promoting balanced learning.

The primary contributions of this study are as follows:

(1) Proposing a multitask state monitoring framework (BiTCN–BiGRU–BiCrossAttention) tailored for electro-hydraulic servo material fatigue testing machines, enabling efficient extraction and fusion of spatiotemporal features;

(2) Introducing a bidirectional cross-attention mechanism into multitask monitoring for such equipment, enhancing inter-task information sharing and reducing negative transfer;

(3) Incorporating the GradNorm strategy into multitask training to dynamically balance task weights, significantly alleviating gradient conflicts and improving overall performance;

(4) Conducting validation experiments on a real fatigue testing machine dataset, demonstrating superior performance of the proposed method in terms of classification accuracy, regression error, and fault localization recall.

## 2. BiTCN–BiGRU–BiCrossAttention Network for Multi-Task State Recognition

**Figure 1:**
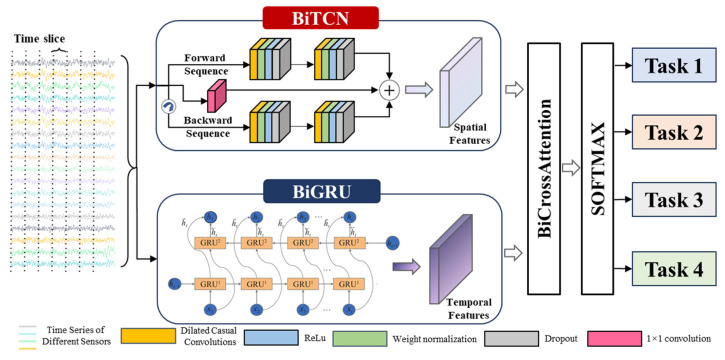
Framework of BiTCN–BiGRU–BiCrossAttention.

The proposed architecture comprises three parallel modules: a Bidirectional Temporal Convolutional Network (BiTCN) for spatial feature extraction, a Bidirectional Gated Recurrent Unit (BiGRU) for temporal dependency modeling, and a Bidirectional Cross-Attention (BiCrossAttention) mechanism for feature fusion. As illustrated in Figure 1, the network comprises three main modules: the BiTCN module, the BiGRU module, and the bidirectional cross-attention module. First, the BiTCN module is applied to perform an initial round of feature extraction, capturing the spatial characteristics of multi-source data. Subsequently, the original data are input in parallel into the BiGRU module to mine the temporal characteristics of multi-source data sequences. Next, the spatial and temporal features obtained above are fused through a bidirectional cross-attention mechanism. Finally, the fused features are fed into a multitask classification module to produce diagnostic labels.

The method proposed in this paper is improved on the BiTCN–BiGRU–Attention proposed by Zhang [23]. Although they have similar network structures, the two have different advantages in feature interaction. BiCcrossAttention establishes cross-modal queries between two different feature Spaces (the spatial channel features of BiTCN and the time series features of BiGRU), which can simultaneously solve two aspects of problems: Which sensor channel is critical at this time step? At what time step does this sensor have the largest amount of information? This kind of bidirectional cross-flow interaction cannot be realized by self-attention or unidirectional cross-attention, and is particularly suitable for electro-hydraulic systems that encode complementary physical knowledge in spatial (multi-sensor) and temporal (dynamic response) dimensions.

### 2.1. BiTCN Module

**Figure 2:**
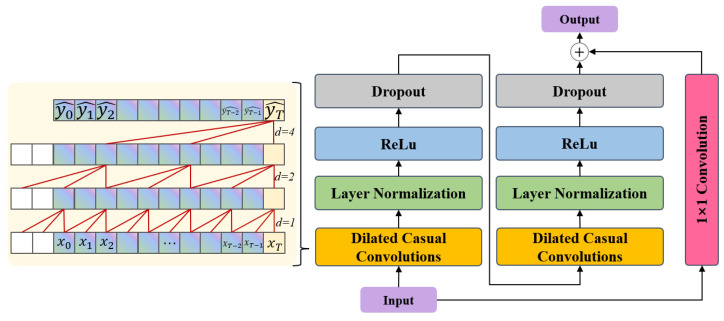
BiTCN Network.

To effectively extract discriminative spatial and local temporal features from the multi-source heterogeneous data collected by the electro-hydraulic servo fatigue testing machine, this model first adopts a Bidirectional Temporal Convolutional Network (BiTCN) as the core feature extractor. This module aims to overcome the limitations of conventional one-dimensional convolution, which has a restricted receptive field and difficulty in capturing long-range dependencies, while enhancing contextual awareness through its bidirectional structure. The computational framework of BiTCN is illustrated in Figure 2.

#### 2.1.1. Dilated Causal Convolution

Two-dimensional convolutional neural networks (CNNs) are typically applied to process data with explicit spatial structures, such as images. For multivariate time-series data (MTS), the data can be considered as a 2D matrix $X\in R^{T\times C}$, where T denotes the number of time steps and C denotes the variable channels (i.e., number of sensors). One-dimensional convolution is well-suit for capturing cross-channel (i.e., spatial) correlations within localized time windows by computing weighted sums across all sensor channels. This operation applies a convolution kernel across all input channels $C_{in}\;$ within each local time window  [t:t+k], computing weighted sums over all sensor inputs. Consequently, the kernel learns local spatial correlation patterns among the signals from different sensors within the time window.

BiTCN employs dilated causal convolution to compute features, as shown in Equation (1),

(1) $$F\left(s\right)=\left(x\times_{d}f\right)\left(s\right)=\sum_{i=0}^{k-1}f\left(i\right)\cdot x_{s-d\cdot i}$$

where d is the dilation factor, k is the filter size, and s−d·i is the direction corresponds to the past. Dilation introduces a fixed step size between adjacent filter taps, thereby enlarging the receptive field without increasing parameter count significantly. When d=1, the dilated convolution reduces to standard convolution.

#### 2.1.2. TCN Bidirectional Processing

Although standard causal convolution is suitable for real-time prediction, bidirectional architecture significantly enhances feature extraction completeness for tasks like state recognition that permit offline analysis using complete historical and future data. Therefore, this module adopts a bidirectional processing flow.

Forward Pass: Processing input sequences $X=(X_{1},X_{2},\ldots ,X_{T})$, dilated causal convolution is performed along the forward temporal direction to capture dependencies from past to current time steps.

Backward Pass: Processing the reversed input sequence $X_{\mathrm{reversed}}=(X_{T},X_{T-1},\ldots ,X_{1})$. Similarly, dilated causal convolution is applied to capture dependencies from the future (end of the sequence) to the current time step.

Feature Fusion: Concatenating the output of Forward Pass ${\mathrm{TCN}}_{\mathrm{forward}}$ and the output of Backward Pass ${\mathrm{TCN}}_{\mathrm{backward}}$ along the feature dimension, forming the final bidirectional spatial feature representation $F_{\mathrm{BiTCN}}$, as shown by Formula (2).

(2) $$F_{\mathrm{BiTCN}}={\mathrm{TCN}}_{\mathrm{forward}}⊕{\mathrm{TCN}}_{\mathrm{backward}}$$

This bidirectional architecture enables the model to simultaneously leverage both complete past and future contextual information when analyzing data at any given moment, thereby achieving more accurate and robust judgments of the current system state.

### 2.2. BiGRU Module

The high-order spatial features $F_{\mathrm{BiTCN}}$ extracted by the BiTCN module, while containing rich cross-sensor correlation information, still require further modeling of their temporal dynamic evolution processes. To capture the long-term dependencies and dynamic behavioral patterns of hydraulic system states along the temporal dimension, the proposed model employs a Bidirectional Gated Recurrent Unit (BiGRU) network as the temporal feature extractor. With its gating mechanisms, the GRU effectively addresses the vanishing gradient problem inherent in traditional Recurrent Neural Networks (RNNs), making it more suitable for learning complex dependencies in long time series.

#### 2.2.1. Basic Principles of Gated Recurrent Units (GRUs)

In GRUs, calculations of update gate $z_{t}$, Reset Gate $r_{t}$, Candidate Hidden State $\tilde{h_{t}}$ and Output Hidden State $h_{t}^{\mathrm{dec}}$ are as shown by Formula (3).

(3) $$\begin{array}{l} \left.r_{t}=\sigma \left(W_{xr}x_{t}^{dc}+W_{hr}(h_{t-1}^{\mathrm{dec}}⊕a_{t})\right)+b_{r}\right)\\ \left.z_{t}=\sigma \left(W_{xz}x_{t}^{dc}+W_{hz}(h_{t-1}^{\mathrm{dec}}⊕a_{t})\right)+b_{z}\right)\\ {\tilde{h}}_{t}=\tanh\left(W_{xh}x_{t}^{dc}+W_{hh}\left(r_{t}⊙(h_{t-1}^{\mathrm{dec}}⊕a_{t})\right)+b_{h}\right)\\ h_{t}^{\mathrm{dec}}=(1-z_{t})⊙h_{t-1}^{\mathrm{dec}}+z_{t}⊙{\tilde{h}}_{t} \end{array}$$

where $W_{xr},W_{hr},W_{xz},W_{hz},W_{xh},W_{hh}$ are weight parameters, $b_{r},b_{z}b_{h}$ are bias parameters, σ is Sigmoid, tanh is the hyperbolic tangent activation function, ⊙ denotes the Hadamard product, ⊕ represents vector concatenation, $x_{t}^{dc}$ represents decoder input, GRU unit output is the hidden state at the current time step $h_{t}^{\mathrm{dec}}$. This hidden state integrates the input information at the current time step with the feature information from previous time steps. Finally, the hidden state is passed to a linear output layer by $h_{t}^{\mathrm{dec}}$.

#### 2.2.2. Bidirectional Architecture and Context Awareness

To fully capture bidirectional dependencies in temporal contexts, this module employs a bidirectional GRU structure. The architecture consists of a forward GRU layer and a backward GRU layer, which process the input sequence and its reversed version, respectively. The forward GRU layer processes the sequence along the chronological direction to compute forward hidden states, while the backward GRU layer processes the sequence in reverse temporal order to generate backward hidden states, ultimately obtaining temporal features $F_{\mathrm{BiGRU}}$.

### 2.3. BiCrossAttention

The core of the bidirectional cross-attention mechanism lies in enabling information interaction between different features. First, linear transformations are applied to spatial features and temporal features to map them to the same dimension $D_{k}$.

In the first stage, spatial features serve as the Query while temporal features function as both Key and Value, yielding $F_{s\to t}$ through computation. This enables spatial features to focus on relevant information within temporal features, thereby excavating the complementary and enhancing effects of temporal features on spatial features. The calculation formula is as follows. First, the attention score $A_{s\to t}$ is computed, as shown in Formula (4).

(4) $$A_{s\to t}=\mathrm{softmax}\left(\frac{Q_{s}K_{t}^{T}}{\sqrt{D_{k}}}\right)$$

where $Q_{s}$ denotes the Query of spatial features, and $K_{t}^{T}$ represents the transpose of the temporal features’ Key.

Subsequently, the weighted sum is calculated based on the attention scores to obtain the fused features, as shown in Formula (5):

(5) $$F_{s\to t}=A_{s\to t}V_{t}$$

where $V_{t}$ represents the Value of the temporal features.

In the second stage, temporal features are used as queries while spatial features serve as keys and values to compute $F_{t\to s}$. This allows temporal features to attend to critical information in spatial features, enabling spatial features to provide feedback to temporal features. Through this bidirectional computation, both types of features can fully absorb useful information from each other, avoiding potential information loss caused by unidirectional computation. Finally, the fused features from both directions are concatenated and linearly transformed to obtain the final fused feature F, as shown in Formula (6).

(6) $$F=\mathrm{Linear}\left(\left[F_{s\to t};F_{t\to s}\right]\right)$$

**Figure 3:**
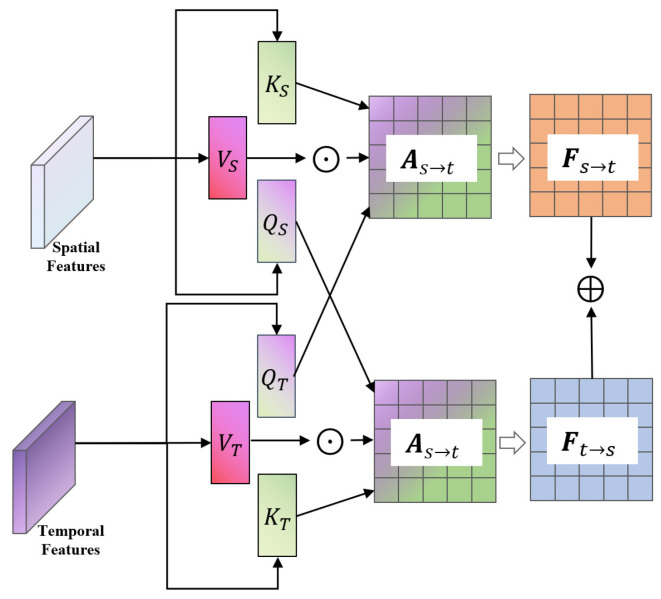
Bidirectional Cross-Attention Framework.

Through the aforementioned cross-attention mechanism, bidirectional interaction and deep fusion between spatial and temporal features are achieved, providing more expressive feature representations for subsequent tasks. The architecture is illustrated in Figure 3.

### 2.4. GradNorm

To effectively balance the losses of multiple tasks and facilitate collaborative learning in the model, this study employs the GradNorm algorithm for multi-task loss calculation. GradNorm dynamically adjusts the weights of individual task losses to maintain consistent gradient magnitudes across tasks during training, thereby preventing any single task from dominating the overall training process. Specifically, GradNorm computes adaptive loss weights based on the magnitude and gradient information of each task’s loss, enabling the model to maintain balanced attention across all tasks during training.

### 2.5. Algorithm Steps (Pseudocode)

This section presents the pseudo-code of the designed algorithm, as shown in Table 1, which is the Notation and Description of Variables in Algorithm 1.


**Algorithm 1: Training Process of Multi-task State Recognition Model for Electro-hydraulic Servo Material Fatigue Testing Machine Based on BiTCN-BiGRU-BiCrossAttention**


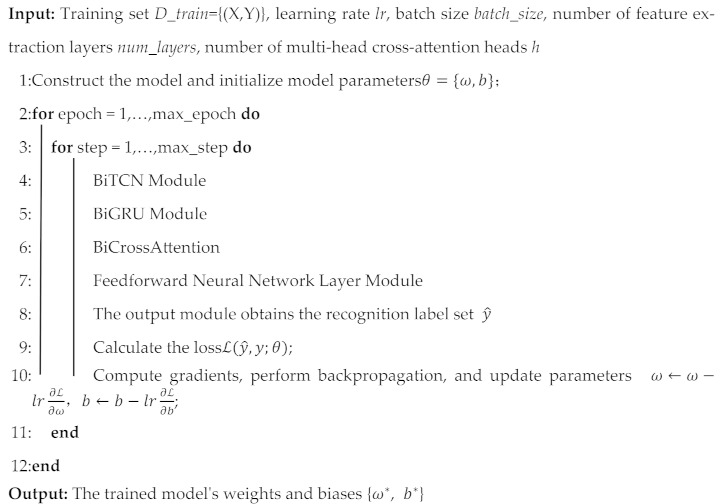



## 3. Case Study

### 3.1. Experimental Study on Multi-Task State Recognition of Electro-Hydraulic Servo Material Fatigue Testing Machine

**Figure 4:**
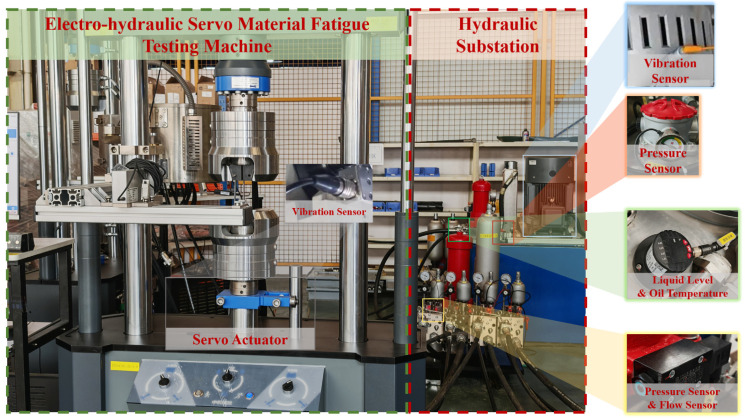
SDZ0100 Electro-hydraulic Servo Material Fatigue Testing Machine Multi-Task State Recognition Experimental Platform.

The state recognition experiment for the testing machine was carried out at a design and manufacturing enterprise specializing in fatigue testing equipment. An SDZ0100 electro-hydraulic servo fatigue testing machine was selected as the monitoring subject. The SD series electro-hydraulic servo fatigue testing machines are capable of simulating complex operational environments and meet the requirements of advanced testing fields such as fatigue and fracture testing for different materials and components. The external view of the experimental platform and some sensor installations are shown in Figure 4.

The proposed BiTCN–BiGRU–BiCrossAttention-based multitask state recognition model for electro-hydraulic servo material fatigue testing machines is primarily applied to four key components: Cooler, Electro-hydraulic Servo Valve, Motor Pump Unit, and Servo Actuator. Data collection is mainly conducted via sensors that capture operational parameters of these components, specifically including multiple pressure sensors, liquid level and oil temperature sensors, flow sensors, and vibration sensors, the sensors configuration is listed as Table 2 below.

To facilitate data analysis, all signals except the vibration signal were resampled at 1 khz. Since parameters such as temperature, flow rate, power, and liquid level do not undergo sudden changes within a short period of time (0.1 s to 1 s), this study uses quadratic interpolation to upsampling them and synchronizes them through hardware triggers. Each 60 s segment contains 60,000-time steps. Raw signals were filtered using a 4th-order Butterworth low-pass filter (cutoff: 200 Hz) to remove high-frequency noise. Features were then standardized per sensor channel (zero mean, unit variance) using statistics computed only on the training set to avoid data leakage.

Model training adopts a supervised learning approach, requiring differentiation between normal and faulty data from the operational dataset of the testing machine, followed by manual labeling. First, Preliminary research with engineering personnel found that transient fault characteristics (such as valve flutter and pressure peaks) usually last less than 5 s. A lower state update frequency in engineering can also lead to lower computational costs. Therefore, a 60 s time window that is convenient for statistics was selected for data annotation. Each segment is then annotated with the operational states of the four key components. A total of 2200 such data segments were collected, forming the experimental dataset.

The detailed state labels and sample counts are presented in Table 3. Since failures of key components are not mutually exclusive and multiple components may exhibit varying degrees of degradation simultaneously, each data segment may correspond to degradation or faults in multiple components. Consequently, each segment is annotated with four separate state labels. Labeling was performed by fatigue testing machine development experts, based on the degradation stages and fault modes characteristic of each key component.

For the servo actuator, state labels were assigned according to the deviation between the target thrust and the measured thrust. For the Motor Pump Unit, labels were determined based on the extent of oil leakage. For the electro-hydraulic servo valve, degradation states were labeled based on the deviation between the target frequency and the measured frequency. For the cooler, labels were assigned based on cooling efficiency to indicate fault conditions in the cooling system. The numeric labels represent different state categories and their corresponding meanings, resulting in the final formation of the experimental dataset.

After obtaining the data, it is necessary to perform a series of preprocessing steps to ensure suitability for model training. The data should be standardized so that all features share the same scale, preventing certain features from exerting an excessive influence on the training process. To ensure robust evaluation and assess the statistical significance of model performance, we performed five independent random splits of the full dataset into training (80%) and validation (20%) sets, stratified by component state labels to maintain class distribution consistency across splits.

### 3.2. Hyperparameter Selection and Training of the BiTCN–BiGRU–BiCrossAttention Multitask State Monitoring Model

The specific architecture of the model is determined by its hyperparameters. The ultimate goal of model training is to adjust these hyperparameters to find the optimal configuration, enabling better learning ability and generalization performance. The main parameters involved in the model are as shown in Table 4:

(1) BiTCN Convolution Kernel: The width of the one-dimensional convolution kernel along the time dimension determines the size of the local time window observed by each convolutional layer. Larger kernels can capture more complex local patterns. TCN typically uses small kernels and relies on dilated convolution to expand the receptive field.

(2) Dilation Factor: One of the core hyperparameters of TCN. It exponentially enlarges the receptive field of the network, allowing deeper layers to capture long-term dependencies without significantly increasing the number of parameters.

(3) Feed-Forward Network Dimension $d_{f}$: The hidden layer dimension of the feed-forward neural network mainly affects the model’s nonlinear representation capability. The value of $d_{f}$ is usually closely related to $d_{\mathrm{model}}$, often set to two or four times $d_{\mathrm{model}}$.

(4) Number of Stacked Feature Extraction Layers N: Determines the depth and representational capacity of the model. Adding more layers enables the model to learn more long-term dependencies and complex feature patterns. For time-series tasks, the number of stacked layers is adjusted according to data complexity and task requirements.

(5) Training-related Hyperparameters: Batch Size: Determines the number of samples used for weight updates in each training step. Optimizer: Algorithm for adjusting model parameters; Adam is commonly used. Learning Rate (lr): Governs the step size for each parameter update. Loss Function: Measures the difference between predicted values and ground truth. For classification tasks, cross-entropy loss is commonly used, quantifying the difference between the output probability distribution and the true labels. Epochs: Number of complete passes through the training dataset during model training.

The model was trained and evaluated using the PyTorch 2.2.1 framework. The hardware configuration of the training environment was as follows: AMD Ryzen 2700X CPU, NVIDIA 2070 GPU, and 16 GB RAM.

**Figure 5:**
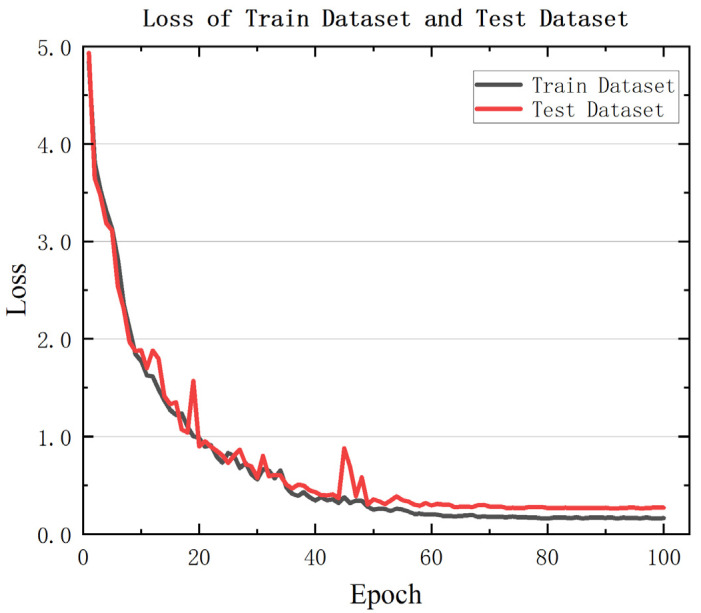
Training and Validation Loss Trends of the Model.

**Figure 6:**
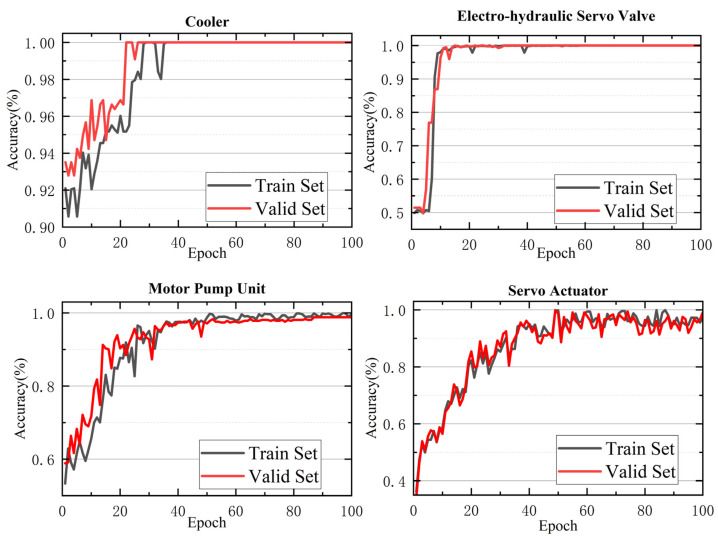
Accuracy of Model Training and Validation Sets.

The trends of training and validation loss values, along with the accuracy trends for recognizing the states of the four key components during the learning process, are shown in Figure 5 and Figure 6.

From the curves in Figure 5, it can be seen that during the model training process, the loss value gradually decreases as the number of iterations increases. After a certain number of iterations, the loss converges, with the training set loss stabilizing below 0.15 and the validation set loss converging below 0.3, indicating good convergence performance.

**Figure 7:**
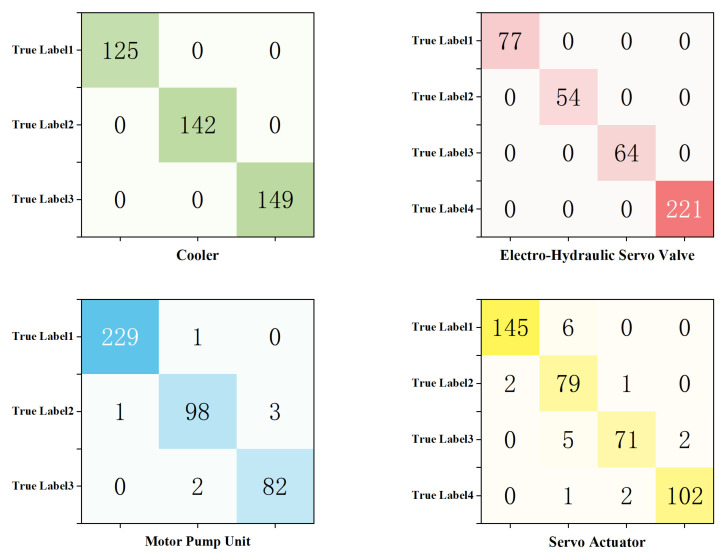
Learning Accuracy of Validation Sets.

From Figure 6, which shows the relationship between classification accuracy (acc) for recognition of the states of the four key components and the number of epochs, it is evident that as the number of iterations increases, the classification accuracy for both the training and validation sets steadily improves and eventually converges. On the validation set, the classification accuracy for the cooler and the electro-hydraulic servo valve both reach 100%, while the accuracy for monitoring the hydraulic pump and the servo actuator states reach 98% and 96%, respectively. To provide a more intuitive view of the correspondence between the model’s predictions and the true labels, confusion matrices for the state detection tasks of the four key components are plotted in Figure 7.

In Figure 7, the horizontal axis represents the predicted classes, and the vertical axis represents the true classes. Each element in the matrix indicates the number of samples corresponding to a specific true–predicted class pair. It can be seen that most elements are concentrated along the diagonal line. The precision, recall, and F1-score of the model on the validation set are presented in Table 5.

The results show that the model achieves excellent performance in the multi-component state monitoring task. On the validation set averaged in five random splits, for the cooler and the electro-hydraulic servo valve, the accuracy, macro-average precision, macro-average recall, and macro-average F1-score all reach 100%, demonstrating exceptionally strong state monitoring capability. For the hydraulic pump, all metrics exceed 97%. Although the performance for the servo actuator is slightly lower, the overall metrics remain at a high level (F1 > 95%), and all task has small standard deviations (<0.5%), proving that the BiTCN–BiGRU–BiCrossAttention-based multitask state monitoring model for electro-hydraulic servo material fatigue testing machines proposed in this chapter achieves outstanding results in state recognition tasks.

## 4. Discussion—Performance Analysis of the BiTCN–BiGRU-–BiCrossAttention-Based Multitask State Monitoring Model for Electro-Hydraulic Servo Material Fatigue Testing Machines

To evaluate the effectiveness of the proposed state monitoring model, a series of comparative experiments were conducted.

### 4.1. Comparison Between Single-Task and Multi-Task Learning—Results and Analysis

The state monitoring model proposed in this chapter is a multi-task learning model. Unlike single-task learning, where each task is modeled independently, multi-task learning performs multiple tasks simultaneously within the same network by sharing features. Therefore, when analyzing performance, it is necessary to compare it with single-task models built using the same backbone architecture.

**Figure 8:**
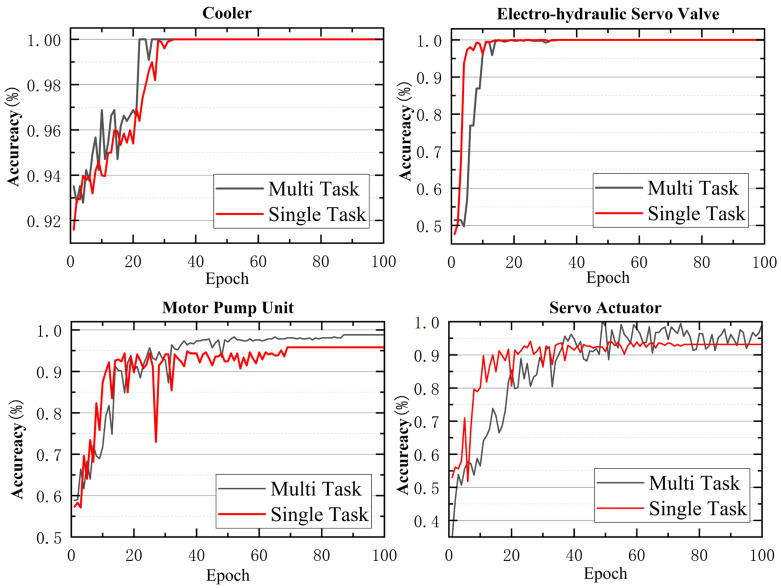
Accuracy Comparison Between Single-Task Learning and Multi-Task Learning.

Under identical hyperparameter configurations, each single-task model was trained individually, and their accuracy on the validation set was computed. The comparison is shown in Figure 8.

It can be found from the figure that the convergence speed of multi-task classification is slightly slower than that of single-task classification. However, multi-task classification does not reduce the accuracy rate compared to single-task classification. This is because useful information is shared among tasks, the regularization effect inhibits overfitting, and the data utilization efficiency is improved.

The precision, recall, and F1-score for single-task classification learning were calculated and presented in Table 6.

From the comparison between single-task and multi-task models, it can be seen that for the state monitoring tasks of the cooler and the electro-hydraulic servo valve, all metrics—accuracy, precision, recall, and F1-score—reach 100% regardless of whether single-task or multi-task learning is used, indicating that the model can accurately predict the statuses of these two components.

For the hydraulic pump and servo actuator, multi-task learning achieves improvements of 3% to 4% across accuracy, precision, recall, and F1-score. This suggests that multi-task learning can capture correlations between tasks, extract deeper-level features, and consequently enhance overall model performance.

From the perspective of lightweight deployment, multi-task learning shares part of the parameters during training, meaning that more tasks can be accomplished with smaller storage and computational resources. Using PyTorch’s torch module to calculate model parameters (Table 7).

It is observed that for a single key component state recognition task, the parameter count difference between single-task and multi-task models is less than 1%, because feature extraction layers share parameters and the difference lies only in the output layers.

However, for monitoring all components of the testing machine, single-task learning requires four independent models (one for each component), whereas multi-task learning only needs one model. As a result, the total computation required for multi-task learning is only 25% of that for single-task learning, significantly reducing computational overhead needed for training and deployment.

These experimental results demonstrate that the multi-task classification design in the output layer of the proposed model offers significant advantages over conventional single-task classification models in terms of both performance and deployment efficiency.

### 4.2. Robustness Experiment Results and Analysis

**Figure 9:**
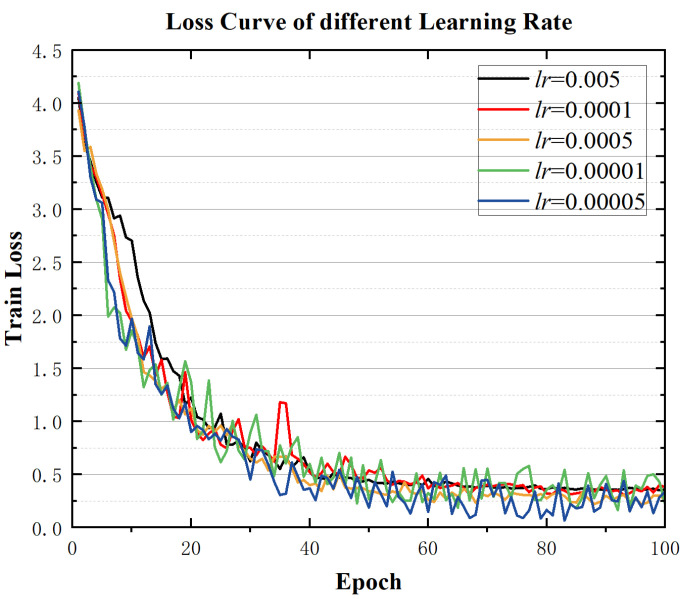
Loss Curves Under Different Learning Rates.

In deep learning, robustness generally refers to a model’s stability under different environmental conditions—specifically, whether the model can maintain a strong performance when parameters or input data vary. Since most hyperparameters are tied to the model architecture, this section focuses on the learning rate *lr* as a critical factor. Learning rates of 0.001, 0.005, 0.0001, 0.0005, and 0.00001 were selected for training the model, and its performance under each setting was compared, as shown in Figure 9.

From Figure 9, it can be seen that under different learning rates, the loss curves consistently show a stable downward trend, with similar convergence speeds and no signs of instability. Because the F1-score offers a comprehensive measure of model performance, the F1-scores obtained under different learning rates are collected in Table 8.

The table shows that the final F1-score on the validation set changes only slightly across different learning rates, demonstrating stable performance. This confirms that the state monitoring model proposed in this chapter exhibits good robustness when faced with varying learning rate configurations.

### 4.3. Ablation Experiments

To systematically evaluate the contribution of each core component in the proposed BiTCN–BiGRU–BiCrossAttention model, a series of comprehensive ablation experiments were designed. These experiments aimed to answer the following three key questions:

① How much does each component (BiTCN, BiGRU, BiCrossAttention) individually contribute to improving final performance?

② What advantages does the proposed bidirectional cross-attention mechanism offer compared to a traditional attention mechanism?

③ Does this hybrid architecture maintain consistent superiority across classification tasks with different characteristics?

**Figure 10:**
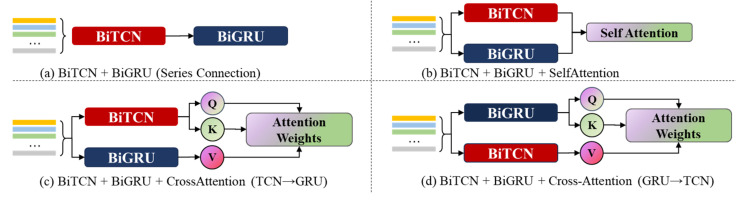
Structural Comparison.

To clearly demonstrate the structural differences among various networks, Figure 10 presents the structural diagrams of 4, 5, 6, and 7 in the ablation experiment.

All ablation experiments were conducted using the same train/validation data split, with fixed random seeds to ensure reproducibility. Macro-average F1-score was adopted as the core evaluation metric, and the results are shown in Table 9.

#### 4.3.1. Analysis of Core Component Contributions

To systematically quantify the impact mechanisms of each architectural component on model performance, this study statistically analyzed the macro-average F1-score and its standard deviation across different model structures in multi-task state recognition, based on five independent stratified random partition experiments. This design not only evaluates the average model performance but also reveals its sensitivity to data partition perturbations through error distribution, thereby reflecting generalization robustness.

Experiments 2–3 reveal that while single feature extractors possess foundational discrimination capabilities, they exhibit significant limitations. BiTCN achieves 98.06 ± 0.80% F1-score on the cooler task, effectively capturing temperature–flow transients induced by abrupt cooling efficiency changes. In contrast, its performance on the servo actuator task drops to 64.12 ± 1.12%, with a notably high standard deviation (>1%), reflecting its limited capacity to model the slow-evolving friction–hysteresis coupling inherent in thrust degradation. In contrast, BiGRU demonstrated superior performance on the actuator task (72.30 ± 0.73%), validating its modeling advantage for long-term dependent signals (e.g., displacement-force hysteresis loops), though it remained inadequate for high-frequency pressure fluctuation-sensitive tasks (e.g., servo valves). When BiTCN and BiGRU were serially fused (Experiment 4), spatial–temporal representation capabilities significantly improved, achieving absolute increases of 5–10% in F1-score across all four component types while narrowing error ranges. This demonstrates that synergistic modeling of local transient responses and global dynamic evolution information is a critical prerequisite for enhancing electrohydraulic system state perception accuracy.

Further introduction of attention mechanisms simultaneously improved performance and stability. Self-attention (Experiment 5) elevated the actuator F1-score to 87.80 ± 1.86% by weighting critical time steps, yet its high standard deviation (1.86%) suggests sensitivity to training set partitioning. This instability in fusion representations may stem from the lack of explicit modeling of cross-channel dependencies. In contrast, unidirectional cross-attention (Experiments 6 and 7) establishes directed interactions between spatial and temporal features, not only boosting actuator performance to approximately 93% but also significantly reducing standard deviation (<1.1%). This demonstrates that explicit cross-modal alignment aids in constructing physically consistent fusion representations. Notably, the control model without bidirectional structure (Experiment 8) approaches the bidirectional version on most tasks but exhibits a slightly higher actuator standard deviation (1.04%) compared to the full model (Experiment 9: 0.93%). This confirms that bidirectional contextual information positively mitigates ambiguity at state boundaries (e.g., distinguishing “mild” from “moderate” thrust reduction).

Ultimately, the proposed model (Experiment 10), after incorporating the GradNorm dynamic loss weighting mechanism, not only achieves optimal average performance (motor-pump unit: 97.72 ± 0.36%, servo actuator: 95.83 ± 0.44%) but also demonstrates exceptional stability—with standard deviations below 0.45% across all tasks. This indicates that GradNorm effectively mitigates multi-task gradient conflicts, enabling thorough optimization for challenging tasks (e.g., actuator degradation recognition) while suppressing performance variance amplification caused by premature convergence in simpler tasks (e.g., cooler failure). In summary, the BiTCN–BiGRU–BiCrossAttention architecture synergistically enhances model classification accuracy and deployment robustness through multi-level spatial-temporal fusion and gradient balancing mechanisms.

#### 4.3.2. Quantitative Analysis of Training Dynamics and Module Contributions

**Figure 11:**
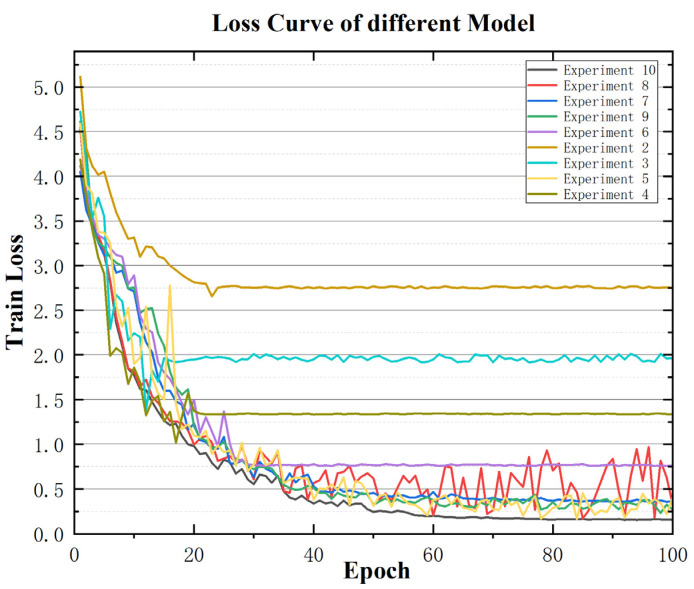
Loss Comparison of Ablation Experiments.

Beyond final F1-scores, we analyze validation loss trajectories of Figure 11 below to quantify the impact of each component on optimization behavior. The full model (Exp.10) achieves the lowest final loss (0.156) and fastest convergence (~60 epochs), whereas removing bidirectional structures (Exp.8) increases final loss by 114% (to 0.333) and slows convergence. Replacing BiCrossAttention with unidirectional variants (Exp.6/7) leads to premature convergence at suboptimal loss levels (~0.76), indicating insufficient feature interaction. Most critically, ablating GradNorm (Exp.9) doubles the final loss (0.363 vs. 0.156) and induces training instability, confirming its essential role in balancing multi-task gradients. These loss dynamics quantitatively explain the F1-score gaps observed in Table 9 and validate our architectural design choices.

#### 4.3.3. Effectiveness Analysis of the Cross Attention Mechanism

To further integrate the output features of BiTCN and BiGRU, we compared different attention mechanisms. The experimental results show that introducing any form of attention improves performance, with the Self-Attention method achieving an average F1-score of 93.39%. However, traditional Self-Attention aggregates information only within its own feature space, failing to fully realize deep interaction between the two modalities. In contrast, unidirectional Cross-Attention (either TCN → GRU or GRU → TCN) allows one sequence’s features to guide attention over another sequence’s features, yielding slightly better performance than Self-Attention. Finally, our proposed BiCrossAttention mechanism establishes a bidirectional, iterative query–response process, enabling deep fusion and complementarity between local features from TCN and global contextual features from GRU. As shown in the last row of Table 1, the complete model achieves the best performance in all tasks, with an average F1-score of 98.38%, significantly outperforming other variants. This demonstrates the critical role BiCrossAttention plays in effectively integrating heterogeneous temporal features.

#### 4.3.4. Consistency Analysis of Multi-Task Performance

We further evaluated the effectiveness of the GradNorm method for multi-task classification in this study. Based on the experimental results and training process, we found that using GradNorm slowed the loss reduction rate for Task 1 and Task 2, synchronizing their loss descent with that of Task 3 and Task 4. Although the loss decreased more slowly, the overall classification accuracy showed clear improvement. This indicates that our model effectively addresses the inherent complex dependency challenges present in multi-task learning scenarios. To more clearly describe the effect of GradNorm in this study, we further statistically analyzed the weight changes in GradNorm, as shown in Table 10.

Data shows that the weight of simple tasks (Cooler, Valve) gradually decreases to prevent them from converging too quickly and dominating the training process, while the weight of difficult tasks gradually increases to provide more learning opportunities. The overall weight will stabilize after 20 to 50 epochs.

### 4.4. Comparative Experiment Results and Analysis

To validate the effectiveness and advantages of the proposed model, comparative experiments were conducted against various existing models. The primary aim was to assess their performance in state monitoring tasks and provide a comprehensive comparison in terms of accuracy, precision, recall, and F1-score.

The following methods were chosen for comparison using the same dataset:

(1) BiTCN–BiLSTM–Attention [23]

This hybrid neural network first uses a bidirectional temporal convolution network (BiTCN) to efficiently extract local features, then applies a bidirectional long short-term memory network (BiLSTM) to capture long-term temporal dependencies, and finally employs an attention mechanism to adaptively weight important time-step features. This combination leverages CNN’s local feature extraction, RNN’s sequence modeling, and Attention’s focus capabilities, making it suitable for complex multivariate time-series prediction and classification tasks.

(2) DSmT-Based Three-Layer Method [24]

Proposed by Xiancheng et al., this method first preprocesses raw signals to build training samples and test samples to be classified. Then, a three-layer hybrid model suitable for hydraulic valves is constructed to detect different fault groups (including actuator coil fatigue and internal valve wear), significantly improving diagnostic accuracy. Finally, classification methods are applied in the first two layers for fault group identification, and in the third layer, the results are fused using Dezert–Smarandache theory (DSmT) for fault type recognition.

(3) Deep Feature Interactive Network (DFINet) [25]

Proposed by Mengqi et al., DFINet measures distribution differences between heterogeneous features within a feature interaction module to separately extract private and shared features from multi-source heterogeneous data. Common fault features are interactively fused while preserving unique private features. Additionally, a global feature fusion module is introduced to adaptively combine surface-level local features and deep abstract features learned from different feature interaction modules.

**Figure 12:**
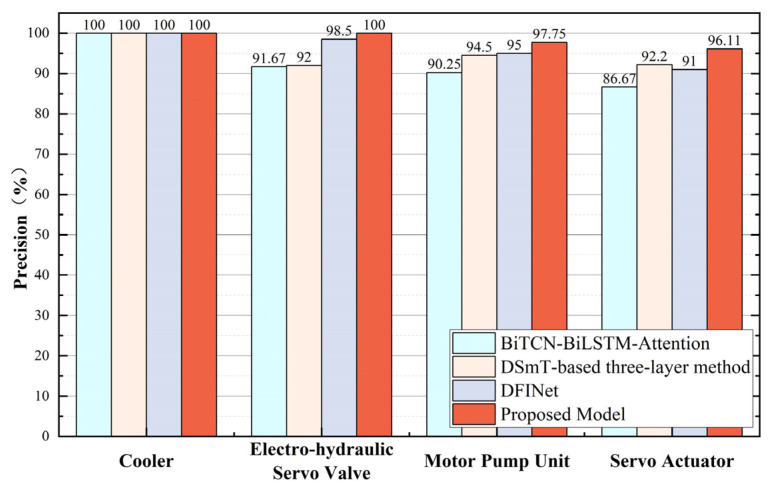
Accuracy Comparison in Comparative Experiments.

For the state monitoring task of key components in the electro-hydraulic servo fatigue testing machine, the final comparison of state detection accuracies on the validation set for each model is shown in Figure 12.

**Figure 13:**
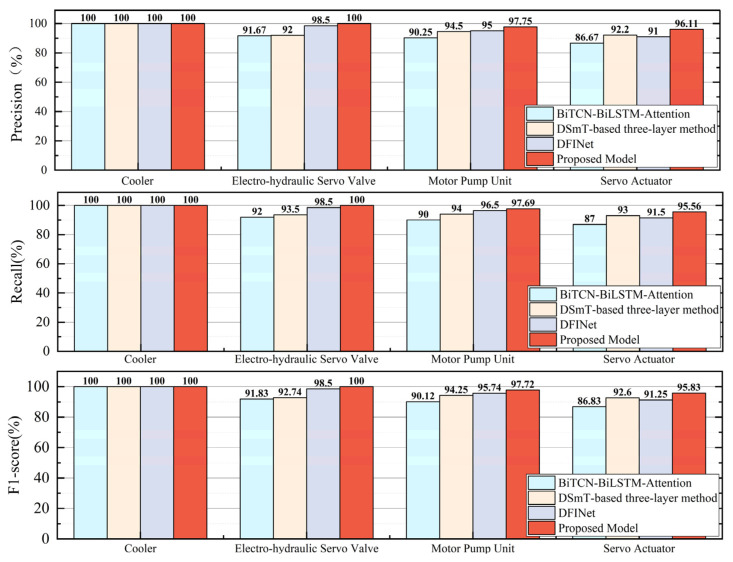
Comparative Evaluation Metrics in Comparative Experiments.

The precision, recall, and F1-score were calculated, and comparative plots were generated for each metric, as illustrated in Figure 13.

From the comparative experiment results, it can be seen that for relatively simple tasks such as cooler status detection, all models achieved 100% accuracy. However, for the status detection tasks of the Electro-hydraulic servo valve, Motor Pump Unit, and Servo Actuator, the model proposed in this chapter achieved significantly higher accuracy, precision, recall, and F1-score than other networks, proving its outstanding performance in state recognition tasks. Comprehensive analysis of evaluation metrics demonstrates that the proposed BiTCN–BiGRU–BiCrossAttention-based state recognition model for electro-hydraulic servo fatigue testing machines exhibits significant advantages over current data-driven methods in both classification performance and multi-task learning architecture design.

### 4.5. Physical Interpretability of the Proposed Architecture

Although the proposed BiTCN–BiGRU–BiCrossAttention framework is data-driven, its design aligns well with key physical characteristics of electro-hydraulic servo systems.

First, the Bidirectional Temporal Convolutional Network (BiTCN) effectively captures local spatiotemporal patterns in multi-sensor signals such as pressure spikes and flow transients. In electro-hydraulic systems, rapid changes in valve current or load often induce high-frequency pressure oscillations and flow surges due to fluid compressibility and pipe elasticity.

Second, BiGRU module is well-suited to represent the slow-varying dynamic behaviors of actuators and pumps. For instance, the servo actuator motion exhibits inertia, friction, and hysteresis, while motor-pump units show thermal drift and leakage accumulation over time. These dynamics manifest as long-range temporal dependencies in displacement, temperature, and vibration signals. By processing sequences in both forward and backward directions, BiGRU leverages full contextual information—akin to how an observer with knowledge of both past loading history and future test trajectory can better infer the current degradation state.

Most importantly, the Bidirectional Cross-Attention mechanism enables explicit interaction between spatial (multi-sensor) and temporal feature representations. This design implicitly encodes the phase relationships and mutual dependencies among physically coupled variables—such as the lag between valve command current and actuator displacement, or the correlation between pump outlet pressure and cooler inlet temperature. Unlike simple concatenation or unidirectional attention, bidirectional cross-attention allows spatial features (e.g., pressure distribution across sensors) to query relevant temporal segments (e.g., peak-load cycles), and vice versa, thereby enhancing physical consistency in multimodal fusion.

While the model remains largely empirical, these architectural choices are not arbitrary; they reflect an implicit encoding of electro-hydraulic system physics through data-informed feature learning. Future work will explicitly integrate governing equations via physics-informed neural networks (PINNs) to further strengthen this interpretability deployment.

## 5. Conclusions

This study proposes and validates a novel multi-state recognition method for electro-hydraulic servo material fatigue testing machines based on spatiotemporal feature fusion and a bidirectional cross-attention mechanism. The key findings, supported by comprehensive experiments and ablation studies, are summarized as follows:

- High-Accuracy Multi-Component Monitoring: The proposed BiTCN–BiGRU–BiCrossAttention model achieves state-of-the-art performance in simultaneous recognition of four critical component states. On a real-world operational dataset from an SDZ0100 testing machine, it attained 100% accuracy and F1-score for both the cooler and electro-hydraulic servo valve, 98.32% accuracy (97.72% F1) for the motor-pump unit, and 96.39% accuracy (95.83% F1) for the servo actuator. This demonstrates its capability for high-precision, concurrent state assessment under complex operating conditions.
- Superiority of Multi-Task Learning: Compared to single-task models with identical backbones, the multi-task framework improves performance by 3–4% for the motor-pump unit and servo actuator tasks. This gain is attributed to the effective sharing of spatiotemporal representations across tasks, which mitigates issues like data imbalance and enhances generalization through implicit regularization.
- Critical Role of Bidirectional Architecture and Fusion Mechanism: Ablation studies confirm that the bidirectional design (BiTCN and BiGRU) is essential for leveraging full contextual information (past and future), leading to more robust state judgments. Furthermore, the proposed bidirectional cross-attention mechanism enables fine-grained, mutual enhancement between spatial and temporal features, outperforming unidirectional or self-attention alternatives by achieving an average F1-score of 98.38%.
- Effective Gradient Balancing and Resource Efficiency: The integration of GradNorm dynamically balances task-specific loss weights during training, successfully alleviating gradient conflicts and promoting stable, balanced learning. In addition, the multi-task model reduces total deployment parameters by approximately 75% compared to four independent single-task models, offering a significantly more lightweight and efficient solution for industrial deployment.
- Strong Robustness: The model exhibits consistent performance under varying hyperparameters (e.g., learning rates) and repeated random sampling, confirming its stability and reliability in practical scenarios.

### Future Prospects and Applications

This study has several limitations. First, the dataset is collected from a specific electro-hydraulic servo fatigue testing machine (SDZ0100) under fixed loading profiles. The model’s generalizability to other machine types or dynamic loading conditions (e.g., random spectrum loading) remains unverified. Second, the proposed method focuses on concurrent state recognition but does not estimate degradation trends or remaining useful life, which are critical for predictive maintenance. Third, although the multi-task design reduces model size by 75%, the computational latency may still be prohibitive for real-time deployment on resource-constrained edge devices.

Building on this foundation, future work will focus on: (1) expanding the dataset to encompass diverse machine types and broader operational conditions (e.g., variable loads and frequencies) to enhance model generalizability; (2) integrating physical knowledge and causal constraints of the hydraulic system into the learning framework to improve interpretability and physical consistency; (3) extending the scope from fault diagnosis to prognostics, such as Remaining Useful Life (RUL) prediction, to enable predictive maintenance; and (4) applying model compression techniques (e.g., pruning and quantization) to facilitate real-time online and edge-device deployment in industrial settings.

## Figures and Tables

**Table 1:** Notation and Description of Variables in Algorithm 1.

Symbol	Description
*X*	Input multi-sensor time-series data (shape: [batch, T, C])
*Y*	Multi-task labels (shape: [batch, 4])
*θ*	Model parameters (weights and biases)
*h*	Number of attention heads in BiCrossAttention
*N*	Number of stacked BiTCN/BiGRU layers

**Table 2:** Sensor Configuration.

Sensors	Deployment Location	Sampling Frequency	Precision
Pressure Sensors 1	Pump Outlet	100 Hz	±1.0%
Pressure Sensors 2	Before the filter	100 Hz	±1.0%
Pressure Sensors 3	After the filter	100 Hz	±1.0%
Pressure Sensors 4	Before the electro-hydraulic servo valve	100 Hz	±1.0%
Pressure Sensors 5	After the electro-hydraulic servo valve	100 Hz	±1.0%
Power parameter acquisition module	The DIN guide rail of the electrical control cabinet	100 Hz	±(1.0%~2.0%)
Flow Sensor	The main oil supply pipeline at the pump outlet	10 Hz	±(0.5~1.0)%
Oil Level Sensor	Hydraulic Reservoir	1 Hz	±(1.0%~3.0%)
Oil Temperature Sensor 1	Main fuel tank return area	1 Hz	±(1.0%~2.0%)
Oil Temperature Sensor 2	Cooler Outlet	1 Hz	±(1.0%~2.0%)
Oil Temperature Sensor 3	Cooler inlet	1 Hz	±(1.0%~2.0%)
Three-axis vibration Sensor 1	Servo actuator (The outer side of the end cover near the piston rod outlet)	10 kHz	±10 g
Three-axis vibration Sensor 2	The bearing housing of the pump casing	10 kHz	±10 g
Three-axis vibration Sensor 3	Electro-Hydraulic Servo Valve	10 kHz	±10 g

**Table 3:** State Labels and Sample Numbers of Key Components.

Components	Fault Description	State Label	State Description	Sample Size
Servo Actuator	Insufficient Hydraulic Rod Thrust	0	Normal thrust	599
		1	Slight thrust reduction	399
		2	Thrust reduction	399
		3	Severe thrust reduction	803
Motor-Pump Unit	Hydraulic Oil Leakage in Motor-Pump Unit	0	Normal	1220
		1	Oil seepage	490
		2	Oil leakage	490
Electro-hydraulic Servo Valve	Valve Switching Lag or Failure	0	Normal switching	1120
		1	Slight switching lag	360
		2	Severe switching lag	360
		3	Switching failure	360
Cooler	Reduction in Cooling Efficiency	0	Normal Cooler	740
		1	Efficiency reduction	730
		2	Approaching failure	730

**Table 4:** Hyperparameter for State Recognition Model of Electro-hydraulic Servo Fatigue Testing Machine.

Hyperparameter	
BiTCN Convolutional Kernel	5
Dilation Factor	8
Feed-Forward Network Dimension $d_{f}$	12
Stacked Layers *N*	6
Optimizer	Adam
Learning rate	0.0005
Batch size	32
Epochs	100
Loss Function	Cross-Entropy Loss

**Table 5:** Accuracy, $P_{\mathrm{Macro}}$, $R_{\mathrm{Macro}}$ and ${F1}_{\mathrm{Macro}}$.

Components	Accuracy	$P_{\mathrm{Macro}}$	$R_{\mathrm{Macro}}$	${F1}_{\mathrm{Macro}}$
Cooler	100 ± 0.00%	100 ± 0.00%	100 ± 0.00%	100 ± 0.00%
Electro-hydraulic Servo Valve	100 ± 0.00%	100 ± 0.00%	100 ± 0.00%	100 ± 0.00%
Motor-Pump Unit	98.32 ± 0.12%	97.75 ± 0.10%	97.69 ± 0.13%	97.72 ± 0.36
Servo Actuator	96.39 ± 0.41%	96.11 ± 0.38%	95.56 ± 0.36%	95.83 ± 0.44%

**Table 6:** Comparison of Single-Task and Multi-Task in acc, $P_{\mathrm{Macro}}$, $R_{\mathrm{Macro}}$ and ${F1}_{\mathrm{Macro}}$.

Components	*acc*		$P_{\mathrm{Macro}}$		$R_{\mathrm{Macro}}$		${F1}_{\mathrm{Macro}}$	
	Single Task	Multi Task	Single Task	Multi Task	Single Task	Multi Task	Single Task	Multi Task
Cooler	100%	100%	100%	100%	100%	100%	100%	100%
Electro-hydraulic Servo Valve	100%	100%	100%	100%	100%	100%	100%	100%
Motor-Pump Unit	95.89%	98.32%	95.81%	97.75%	95.79%	97.69%	95.31%	97.72%
Servo Actuator	94.25%	96.39%	92.80%	96.11%	92.67%	95.56%	92.25%	95.83%

**Table 7:** Model Parameter Quantity.

Training Method	Cooler	Electro-Hydraulic Servo Valve	Motor-Pump Unit	Servo Actuator	Total
Single Task	4,033,899	4,033,947	4,033,665	4,033,978	12,101,511
Multi Task	408,816				408,816

**Table 8:** Experimental Results of F1-score for Models with Different Learning Rates.

lr	Cooler	Electro-Hydraulic Servo Valve	Motor-Pump Unit	Servo Actuator
0.005	100%	100%	95.75%	93.42%
0.0001	100%	100%	97.83%	94.40%
0.0005	100%	100%	98.20%	95.61%
0.00001	100%	100%	98.92%	94.84%
0.00005	100%	100%	97.47%	94.69%

**Table 9:** F1-Score in Ablation Experiments.

Experiment Number	Model	Cooler (%)	Electro-Hydraulic Servo Valve (%)	Motor-Pump Unit (%)	Servo Actuator (%)
1	LSTM (Baseline)	94.14 ± 0.77	94.76 ± 0.20	76.42 ± 0.54	67.94 ± 1.81
2	BiTCN only	98.06 ± 0.80	96.40 ± 0.28	76.76 ± 2.00	64.12 ± 1.12
3	BiGRU only	96.07 ± 0.60	95.54 ± 0.55	80.12 ± 0.72	72.30 ± 0.73
4 (Figure 10a)	BiTCN + BiGRU (Series Connection)	98.38 ± 0.82	98.50 ± 0.41	87.16 ± 1.10	78.50 ± 1.76
5 (Figure 10b)	BiTCN + BiGRU + Self-Attention	97.53 ± 1.16	98.90 ± 0.45	90.30 ± 0.98	87.80 ± 1.86
6 (Figure 10c)	BiTCN + BiGRU + Cross-Attention (TCN→GRU)	100 ± 0.00	99.20 ± 0.68	93.66 ± 0.94	93.10 ± 0.45
7 (Figure 10d)	BiTCN + BiGRU + Cross-Attention (GRU→TCN)	100 ± 0.00	99.46 ± 0.45	93.76 ± 1.28	92.90 ± 1.09
8	TCN + GRU + CrossAttention	100 ± 0.00	99.86 ± 0.18	94.44 ± 1.45	92.80 ± 1.04
9	BiTCN + BiGRU + BiCrossAttention (Without Grad Norm)	100 ± 0.00	99.66 ± 0.34	95.58 ± 0.78	93.60 ± 0.93
10	BiTCN + BiGRU + BiCrossAttention (Proposed Model)	100 ± 0.00	100 ± 0.00	97.72 ± 0.36	95.83 ± 0.44

**Table 10:** Weight Changes in GradNorm.

Epoch	*w*1 (Cooler)	*w*2 (Valve)	*w*3 (Motor Pump)	*w*4 (Actuator)
1	1.0	1.0	1.0	1.0
10	0.7	0.75	1.2	1.35
30	0.6	0.65	1.3	1.45
60	0.55	0.6	1.35	1.5
100	**0.5**	**0.55**	**1.4**	**1.55**

## Data Availability

The data that support the findings of this study are available from the corresponding author upon reasonable request.

## Abbreviations

BiTCN	Bidirectional Temporal Convolutional Network
BiGRU	Bidirectional Gated Recurrent Unit
BiCrossAttention	Bidirectional Cross-Attention Mechanism
MTL	Multi-Task Learning
TCN	Temporal Convolutional Network
GRU	Gated Recurrent Unit
RUL	Remaining Useful Life
SDZ0100	Equipment series number of the electro-hydraulic servo fatigue testing machine used in experiments
